# Power Frequency Breakdown Properties of LDPE-Doped Inorganic Nanoparticles

**DOI:** 10.3390/molecules30091914

**Published:** 2025-04-25

**Authors:** Yujia Cheng, Guang Yu

**Affiliations:** Mechanical and Electrical Engineering Institute, University of Electronic Science and Technology of China Zhongshan Institute, Zhongshan 528400, China; chengyujia@zsc.edu.cn

**Keywords:** nanocomposites, LDPE, insulation materials, breakdown field strength

## Abstract

Although polyethylene is widely used in electrical insulation, it does not possess dielectric properties. It is therefore desirable to develop insulation materials with excellent dielectric properties. In this study, low-density polyethylene (LDPE) was used as a matrix resin, while MgO, wollastonite, and montmorillonite (MMT) were employed as inorganic nano-additives. Three composites were prepared using the boiling–melt blending approach. Power frequency breakdown tests were performed on the original LDPE and on the prepared nanoparticle/LDPE composites. Upon combination with the Weibull distribution, the breakdown test results revealed that the addition of these nano-additive particles to the LDPE matrix increased the breakdown field strength of the material. The highest breakdown field strength for the nano-MgO/LDPE composite was obtained using a MgO loading of 0.5%. Notably, the obtained value was 1.8% higher than that of the pure LDPE. In addition, the highest breakdown field strength for the nano-wollastonite/LDPE composite was obtained using a wollastonite loading of 1% (7.48% higher than that of pure LDPE). Similarly, the highest breakdown field strength of the nano-MMT/LDPE composite was obtained using an MMT loading of 3%, giving a value that was 6.67% higher than that of the pure LDPE.

## 1. Introduction

When cable insulation materials become damp or suffer from excessive temperatures and voltages, their insulating capacities and conductivities are lost; this phenomenon is known as insulation breakdown. In many instances, insulation breakdown is caused by mechanical, thermal, or environmental factors, wherein the breakdown mechanism is closely related to the mechanical and thermal properties of the materials [1]. In recent years, the importance of ultrahigh-voltage (UHV) transmission has gradually increased, which has led to simultaneous increases in the voltage levels and capacities of high-voltage equipment [2]. However, UHV transmission cables are known to encounter several significant challenges, leading to insulation failure and operational power faults [3]. To address such issues, it is necessary to enhance the properties of insulation materials in electrical equipment to ensure the safe operation of high-voltage equipment. More specifically, the insulation materials employed in cables should possess a high breakdown strength [4].

Currently, high molecular weight polymers are commonly used as electrical insulation materials, including LDPE, which possesses a high insulation resistance, low losses, and is easily processed [5]. However, the breakdown field strength of pure LDPE is not sufficiently high, thereby limiting its application scope. LDPE is a semi-crystalline high molecular weight polymer that possesses consecutive defect traps and plays a key role in the heterogeneous nucleation of polymers to promote spherulite formation [6]. In such systems, the presence of abundant interfaces between the crystalline and non-crystalline phases generates additional defect traps, which ultimately enhances the composite microstructure [7]. Notably, the addition of nano-ZnO and nano-MgO particles to LDPE alters its charge injection and transport properties and leads to improvements in the breakdown resistance and electrical treeing [8]. This can be accounted for by considering that inorganic nanoparticles are known to exhibit large specific surface areas, small particle distances, and high surface energies. Thus, upon the introduction of a small number of nanoparticles into the LDPE matrix, the electrical properties are significantly enhanced.

In a previous study, Mantarci et al. employed organically modified montmorillonite (O-MMT) to enhance the breakdown field strength of an O-MMT/EP composite. Upon the incorporation of 3% O-MMT, the highest breakdown field strength was reached, representing a 1.47-fold increase compared to that of the pure EP [9]. This was attributed to the material interface structure, trap quantity, and particle dispersion. In addition, Cheng et al. used different coupling agents to modify the surfaces of ZnO nanoparticles (NPs, ~40 nm diameter), and the corresponding nano-ZnO/LDPE materials were prepared using the melt blending approach. They found that the addition of ZnO NPs increased the breakdown field strength of the original LDPE. Following the modification of the ZnO NPs with the silane coupling agent KH570, the highest composite breakdown field strength was achieved with a ZnO loading of 3%, giving a value that was 11% higher than that of the pure LDPE. In another study, Long et al. subjected nano-MMT/LDPE composites to breakdown tests and found that the addition of nano-MMT NPs enhanced the breakdown strength of the original LDPE, giving the greatest effect at a nano-MMT loading of 3% [10]. Furthermore, Liu explored the effects of adding TiO_2_ and Al_2_O_3_ on the breakdown properties of fluorinated ethylene propylene (FEP) [11]. Upon increasing the TiO_2_ content, the breakdown strength of the nano-TiO_2_/FEP initially increased and then decreased. Moreover, Wang found that that the addition of nano-wollastonite enhanced the polymer breakdown strength of an impregnated varnish [12]. Further, Vinothkumar investigated the effects of different Al_2_O_3_ contents on the breakdown properties of Al_2_O_3_/polyimide (PI). It was found that an Al_2_O_3_ content of 5 wt% led to the greatest breakdown field strength for the Al_2_O_3_/PI composite [13]. Additionally, Hussain et al. explored the effects of different NPs on the dielectric strengths of PIs. At loadings <10 wt%, the addition of nano-SiO_2_ and nano-Al_2_O_3_ particles enhanced the dielectric strength of the pure PI. However, nano-BaTiO_3_, nano-SiC, nano-TiO_2_, and nano-ZnO particles led to reduced dielectric strengths due to the high dielectric constants and uneven distributions of the NPs [14].

It has been previously reported that LDPE and inorganic nanoparticles can form composites at the nano-scale, resulting in a large contact area at the interface between the two species [15]. This differs from conventional interfacial bonding, ultimately leading to variations in the material macro-properties, such as the dielectric properties [16]. Nanocomposites differ from traditional single-component or single-phase nanomaterials, since at least one phase of a one-dimensional scale reaches the nano-scale level. As previously reported, upon the uniform dispersion of NPs into a polymer matrix, the specific surface area of the NPs is increased and the interface effect formed by the polymers becomes more obvious. As a result, the physical properties of the resulting nanocomposites are superior to those of single matrices or nanoparticles, thereby indicating the importance of investigating such nanocomposites.

Common methods of polymer/inorganic nanocomposite preparation include the sol–gel, intercalation compounding, and melt blending methods. Among these, melt blending is the most commonly used approach for nanocomposite preparation due to its simple operation and good blending effect. In this method, a matrix/NP composite is prepared in a stepwise manner, allowing careful control of the NP morphology and size [17]. However, NP agglomeration can easily occur via this route and it is difficult to achieve a uniform particle dispersion. Thus, prior to blending, it is necessary to modify the NP surface to promote uniform dispersion within the matrix. Consequently, polymers that are molten under high-temperature heating conditions have been prepared. During melt blending, the materials are blended using mechanical shear stress, either via Banbury blending (i.e., in an enclosed mixing chambers) or open blending (i.e., open to the atmosphere).

In this study, the breakdown properties of various inorganic nanoparticle/LDPE composites are investigated. More specifically, LDPE is used as the matrix resin, while nano-MgO, nano-wollastonite, and nano-MMT are employed as the inorganic nano-additives to produce nano-MgO/LDPE, nano-wollastonite/LDPE, and nano-MMT/LDPE composites via the melt blending approach. Subsequently, the composite breakdown strengths of the prepared composites are verified by means of power frequency breakdown testing. Moreover, the effects of these inorganic NPs on the electrical characteristics of LDPE are investigated.

## 2. Results

### 2.1. Breakdown Mechanism of Polymer Materials

Polymers are widely used as cable insulation materials, wherein the operational reliability of the cable is largely determined by the normal operation of the insulation material. Upon increasing the rated voltage of a power system, the reliability requirements for the power supply of the system are increased. In recent years, high-voltage technologies have been hindered by the electric power industry and their applications have been limited due to a lack of suitable insulation materials for high electric field applications. Consequently, it is necessary to investigate the breakdown mechanisms of polymers under an external electric field.

Considering the polymer breakdown mechanism under an electric field, the electron avalanche breakdown and free-volume breakdown theories are frequently employed, wherein polymers in the condensed state can be considered as highly compressed gases. Experimental results from Ieda confirmed the electron avalanche breakdown mechanism at low temperatures. More specifically, electron avalanche breakdown is defined as a pure electronic breakdown process that occurs in polymers at low temperatures, wherein the breakdown strength is inversely related to the sample thickness and directly related to the temperature. In this process, the gap breakdown delay is <1 ns and the free volume can accumulate kinetic energy from electrons under an electric field. As a result, a larger free volume leads to a longer electron mean free path, a higher ionization probability after electron collision, and a low breakdown field strength.

Although dielectric breakdown incorporates the gas, liquid, and solid phases, solid dielectric breakdown was explored in detail in the current study due to the use of nanocomposites. Notably, the breakdown strength is an important parameter for determining the dielectric strength of an insulation material and the breakdown strength of a solid dielectric is higher than that of a gas or liquid dielectric. According to the energy type, the concept of solid dielectric breakdown incorporates electric breakdown, thermal breakdown, and electromagnetic breakdown. The principle of electric breakdown is relatively simple and can be used to characterize the intrinsic insulation characteristics of dielectrics. Therefore, electric breakdown is employed for the purpose of this study. During the electric breakdown process, the electrical charges in the dielectric accumulate continuously because of the effect of the high electric field, from which a large amount of electric field energy accumulates in the dielectric material. Subsequently, the electric field energy is converted into heat and chemical energy, which alter the internal structure of the material, and can lead to permanent irreversible damage. The theory of electric breakdown is based on the theories of impact ionization, avalanche breakdown, and charge traps, which exhibit short breakdown times and high breakdown strengths that are related to the degree of electric field distortion and not to the temperature. The related theoretical explanations are summarized in Table 1.

### 2.2. Interface Model Structure

Various research groups have investigated the incorporation of different NPs into polymer matrices and have examined their effects on the dielectric properties of the nanocomposites using related physical models and the dielectric theory. For example, to provide insight into the properties and phenomena of nanodielectrics, Tanaka proposed a multicore model that was based on colloid chemistry, polymer chemistry, and solid-state physics. In the case of polymer composites, such materials are obtained through direct contact at the interfaces. Since the degree and strength of interfacial bonding directly affect the electrical properties of the resulting polymer composites, it is desirable to investigate the structures, properties, and formation mechanisms of such interfaces.

**Figure 1:**
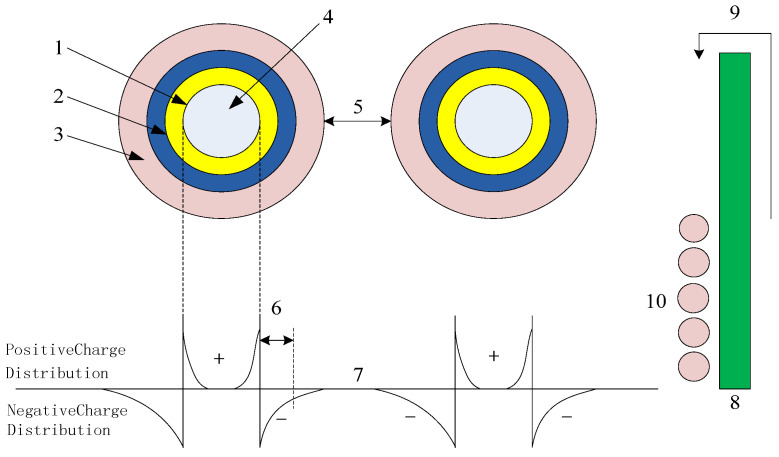
Multi-core model representing a nanocomposite incorporating spherical inorganic NPs within a polymer matrix. (1) Dense first layer, (2) second layer containing deep traps, (3) third layer containing large amounts of free volume, deep ion traps, and shallow electron traps, (4) NP, (5) interparticle distance, (6) Debye shielding distance, (7) NP overlap in the third layer, (8) charge accumulation at the nano filler on the electrode surface, (9) emission of thermoelectrons via the Schottky effect under a high electric field, and (10) changes in charge injection through the neutralizing effect.

A multicore model of spherical inorganic NPs incorporated into a polymer is presented in Figure 1.

More specifically, Figure 1 shows the effect of the nanoparticle addition on the polymer matrix. This model is based on the interactions between interfaces, which possess a three-layer structure. The first layer is the transition region, which is composed of a thin nanometer-scale medium. It acts as a coupling agent that connects the matrix and the NPs. The second layer is the binding layer. This layer has a regular shape that is formed by the action of the polymer chain segment with the first layer. Consequently, this layer represents the interface region between the NPs and the matrix, and its thickness is ~9 nm. Notably, this thickness depends on the strength of the interactions found at the organic–inorganic interface. The third layer constitutes a binding layer exhibiting dielectric bilayer stacking. This layer is similar to a polymer in terms of its chain segment structure, migration rate, free volume, and crystallinity. The second and third layers are relatively loose, tightly coupled, and exhibit a far-field effect, and the layer thickness tends to increase upon moving outward from the NP core. Since the macroscopic- and micro-scale morphologies of different materials cause differences in the layer thicknesses within the multi-core model, the intensity of the far-field effect caused by each composite is different.

Many breakdown phenomena can be observed in this interface model. Considering that the composite interface is an interaction region with a radius of <20 nm, the percolation threshold in the active region is altered upon the addition of NPs and, consequently, the properties of the nanocomposite are changed. More specifically, the incorporation of NPs tends to increase the dielectric strength of the composite. This can be attributed to the acceleration of free electrons within the dielectric material under the effect of an external electric field. These electrons can then infiltrate the third layer and, owing to Coulombic forces, free electrons are evacuated, thereby increasing the breakdown field strength of the dielectric. In addition, the breakdown strength depends primarily on the free volume. In the third layer of the free-volume model, the free volume decreases if defects exist in the nanoparticle structure, ultimately leading to an increase in the breakdown strength.

### 2.3. Weibull Analysis of the Breakdown Field Strength

The Weibull distribution is a widely used and reliable distribution method that is considered to be suitable for analyzing the breakdown field strengths of insulation materials. More specifically, the Weibull distribution reflects the electric strength and time of the insulation material under a certain electric field intensity. Thus, Weibull analysis of the breakdown field strength was performed through the application of the Weibull distribution to the fatigue tests. Following the development of the corresponding Weibull model, large quantities of failure data were implemented. Subsequently, the Weibull distributions were determined and the sample breakdown probability was obtained using Equation (1):

(1) $$P(E)=1-\exp(-{\left(\frac{E}{E_{0}}\right)}^{\beta })$$

where *P* is the probability of sample breakdown, *E* is the simple breakdown field strength, *β* is the shape parameter used to characterize the data dispersion, and *E*_0_ is the scaling parameter of the electric field strength at *P* = 63.2%. Equation (1) can be converted into Equation (2), which represents the relationship between ln(−ln(1−*p*)) and *β*(ln*E*−ln*E*_0_) in a rectangular coordinate system.

(2) $$\ln(-\ln(1-p))=\beta (\ln E-\ln E_{0})$$

According to the 930-2004 standard [18], the probability of a sample being broken down can be calculated using Equation (3):

(3) $$P_{i}=\frac{i-0.44}{n+0.25}\times 100\%$$

where *i* represents the order of the tested samples and *n* denotes the total number of tests performed (i.e., *n* = 15). In addition, the 90% confidence interval was calculated using the 930-2004 Confidence Interval Table.

### 2.4. Power Frequency AC Breakdown Tests

**Figure 2:**
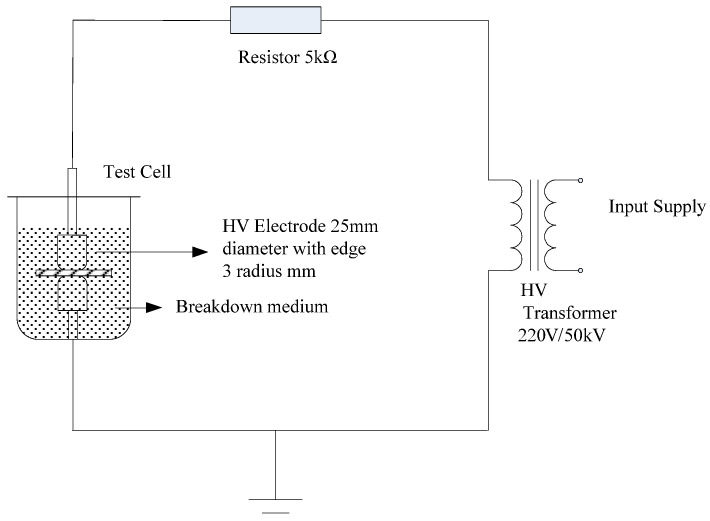
Experimental setup used to perform the breakdown tests.

The experimental setup employed to perform the power frequency AC breakdown tests is presented in Figure 2. During these tests, the AC pressure was increased at rate of 1 kV/s. When the transformer emitted a short-circuit signal, the pressure increase immediately stopped and the samples were broken down. The applied voltage at which this point was reached was recorded as the sample breakdown voltage. According to the breakdown voltage (*U*) and the average thickness of the breakdown points (*d*), the sample power frequency AC breakdown field strength (*E*) was calculated according to the equation *E* = *U*/*d*. In addition, the Weibull distribution curve was obtained using MINTTAB R2022a statistical software.

The breakdown tests were performed using nano-MMT/LDPE, nano-MgO/LDPE, and nano-wollastonite/LDPE samples containing additive loadings of 0, 0.5, 1, 3, and 5%. Each sample possessed a thickness of ~100 µm and was tested at 20 points to determine its average breakdown field strength. To eliminate internal electrical charges during the preparation process, the samples were initially subjected to a short-circuit treatment. Subsequently, these samples were heated at 80 °C in an oven for 12 h prior to cooling naturally to room temperature to eliminate the effects of stress during material mixing and composite preparation.

Although material insulation strength tests are generally performed in an actual operating medium, this medium can easily cause a flashover or intense discharge, leading to serious discrepancies in the test results. To prevent this, the entire electrode system was immersed in filtered cable oil as a liquid medium. More specifically, the cable oil was filtered through filter paper to give the pure medium use in the breakdown tests.

**Figure 3:**
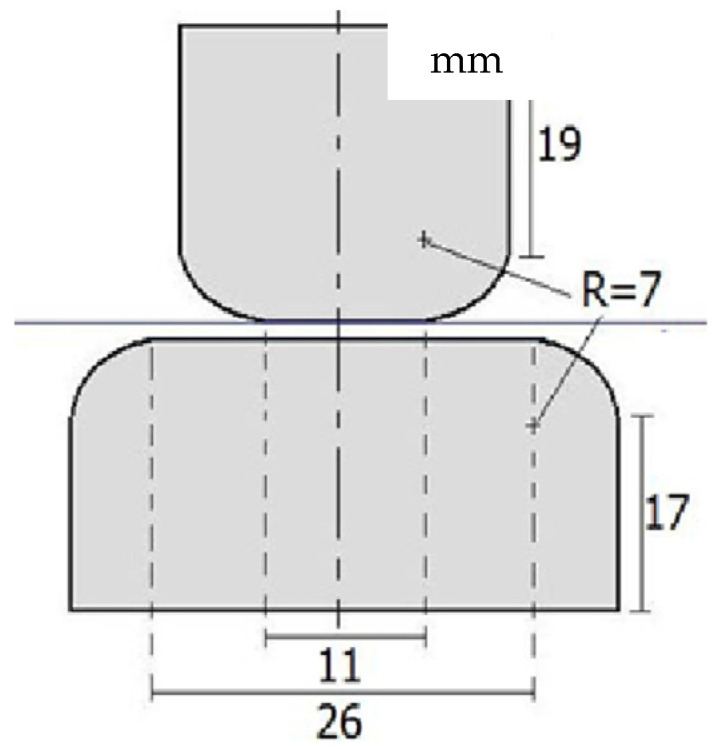
Schematic representation of the electrodes employed during the breakdown test.

The electrodes employed during these tests (see Figure 3) were maintained clean to avoid pit generation. When the upper and lower electrodes were unequal in size, the larger electrode was connected to the ground strap of the transformer. In the absence of ground straps, the larger electrode was connected to the terminal close to the Earth potential. According to the regulations, the electrodes were composed of cylindrical stainless steel, and the electrode surfaces were smooth. Prior to carrying out the experiments, the electrode surfaces were polished with metallographic sandpaper. During testing, two electrodes were placed concentrically to prevent marginal discharge.

### 2.5. Effect of the NP Content on the Composite Breakdown Properties

**Figure 4:**
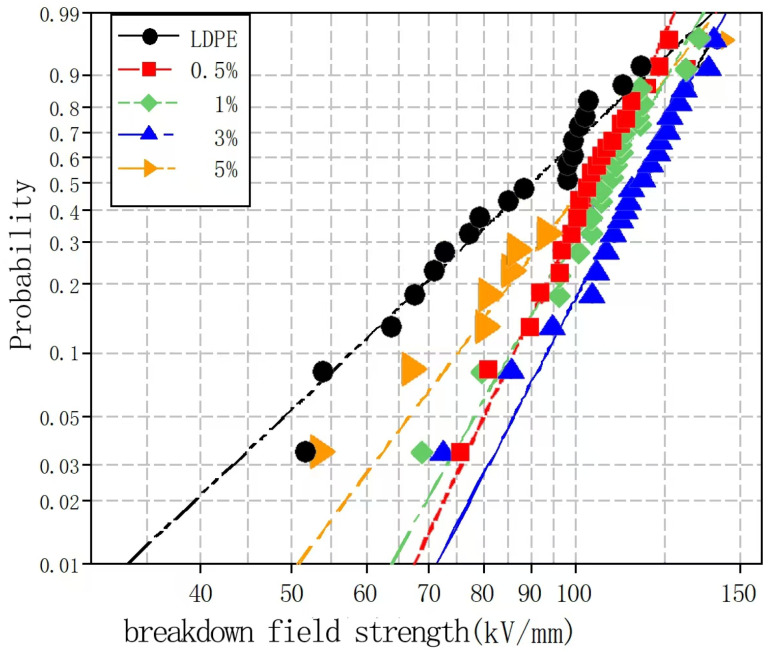
Weibull distributions of the different nano-MMT/LDPE composites during the power frequency breakdown tests.

**Figure 5:**
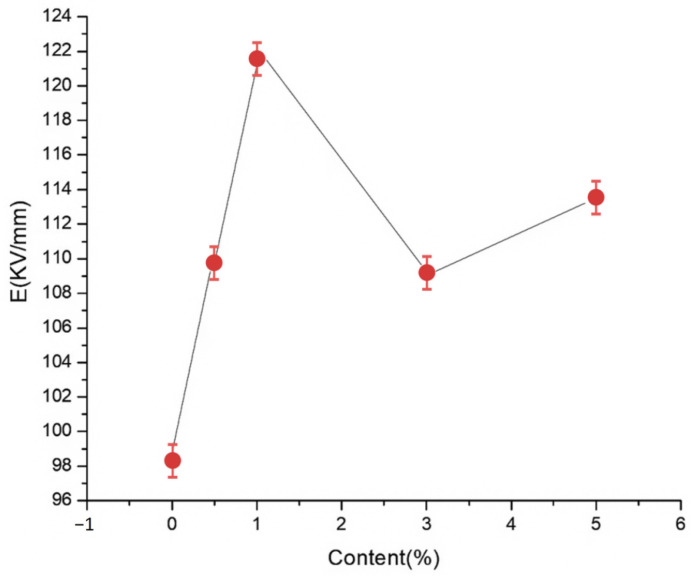
Weibull parameters for the different nano-MMT/LDPE composites during the power frequency breakdown tests.

To explore the effects of different nano-MMT particle contents on the composite breakdown field strength, nano-MMT/LDPE specimens containing 0.5, 1, 3, and 5% MMT were subjected to a breakdown test. The experimental data were analyzed using the Weibull distribution, and the results are presented in Figure 4 and Figure 5.

Figure 4 and Figure 5 show the Weibull distributions and Weibull parameters obtained for the nano-MMT/LDPE composites during the power frequency tests. It can be seen that upon increasing the MMT content, the breakdown field strength initially decreased, then increased, and finally decreased again. The highest breakdown field strength was recorded for an MMT loading of 3%, and the resulting value was 19.14% higher than that of the pure LDPE. Upon increasing the MMT content to 5%, the breakdown field strength of the nano-MMT/LDPE composite reached its lowest value, although this was still 6.7% higher than that of the pure LDPE. These results can be accounted for by considering that at a low MMT loading of 1%, a non-uniform dispersion of NPs was obtained and the distance between NPs was large. Consequently, the interface structure was rather loose and electrons were able to migrate easily. This is beneficial for the formation of conductive pathway, and so the breakdown field strength increased slightly. However, at an MMT loading of 3%, the NPs occupied the amorphous region of the polymer, reducing the amount of free volume and increasing the breakdown field strength. At a higher loading of 5%, significant NP accumulation was observed in the matrix and an uneven interfacial structure was formed. As a result, more abundant free charges accumulated at the interface, electric field distortion occurred, and the breakdown field strength was reduced. In addition, at higher MMT contents, the distance between NPs was reduced and the patterns of the transition region overlapped, thereby lowering the potential barrier to promote carrier migration. As a result, the breakdown field strength of sample 12 was found to be lower than that of sample 11.

**Figure 6:**
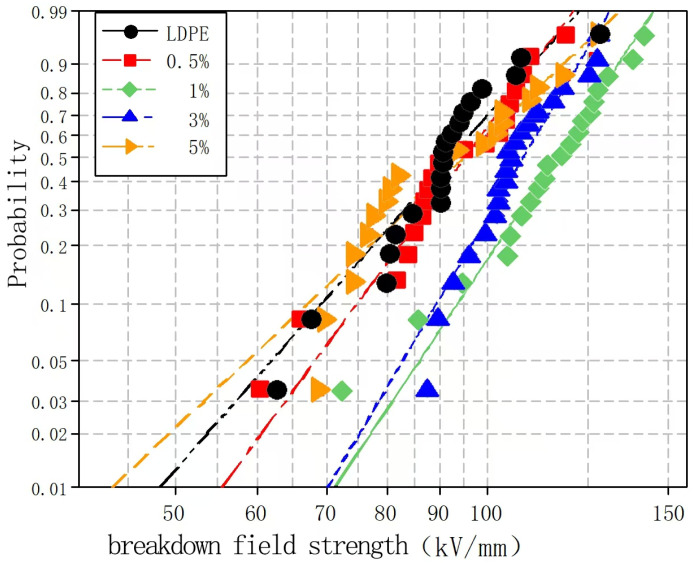
Weibull distributions of the different nano-wollastonite/LDPE composites during the power frequency breakdown tests.

**Figure 7:**
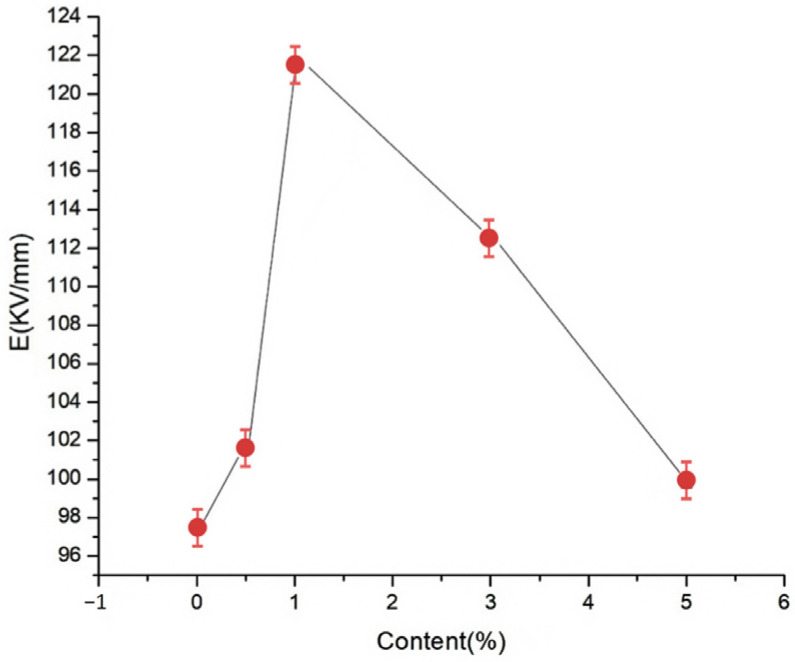
Weibull parameters for the different nano-wollastonite/LDPE composites during the power frequency breakdown tests.

To explore the effects of different nano-wollastonite particle contents on the composite breakdown field strength, nano-wollastonite/LDPE composites containing 0.5, 1, 3, and 5% wollastonite contents were subjected to breakdown testing. Subsequently, the experimental data were analyzed using the Weibull distribution, and the results are presented in Figure 6 and Figure 7.

Figure 6 and Figure 7 show the Weibull distributions and Weibull parameters obtained for the nano-wollastonite/LDPE composites during the power frequency tests. It can be seen that upon increasing the wollastonite content, the breakdown field strength of the nano-wollastonite/LDPE initially decreased, then increased, and finally decreased again. The highest breakdown field strength was recorded for a wollastonite content of 1%, giving a value 19.9% higher than that of the pure LDPE. In contrast, the lowest breakdown field strength was obtained at a wollastonite content to 5%, although this value was still 7.5% higher than that of the pure LDPE. Notably, using a wollastonite content of 0.5%, the NPs were dispersed non-uniformly, the distance between the NPs was large, and the structure of the interface area was relatively loose. Consequently, electrons migrated easily, which was beneficial for the formation of conductive paths, and this led to a slight increase in the breakdown field strength. At a higher wollastonite content of 1%, the NPs became uniformly dispersed, resulting in a superior interfacial structure. In this case, the electronic transitions must overcome the higher carrier, which ultimately restrains directional electron migration. As a result, the number of free electrons in the interfacial region decreased, and the breakdown field strength clearly increased. At a higher wollastonite content of 5%, significant NP accumulation was observed in the matrix. This led to the generation of an uneven interfacial structure, which reduced the breakdown field strength of the composite.

**Figure 8:**
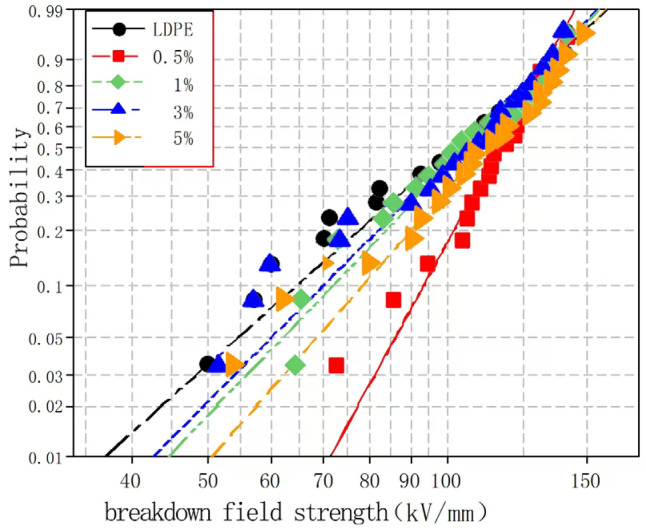
Weibull distributions of the different nano-MgO/LDPE composites during the power frequency breakdown tests.

**Figure 9:**
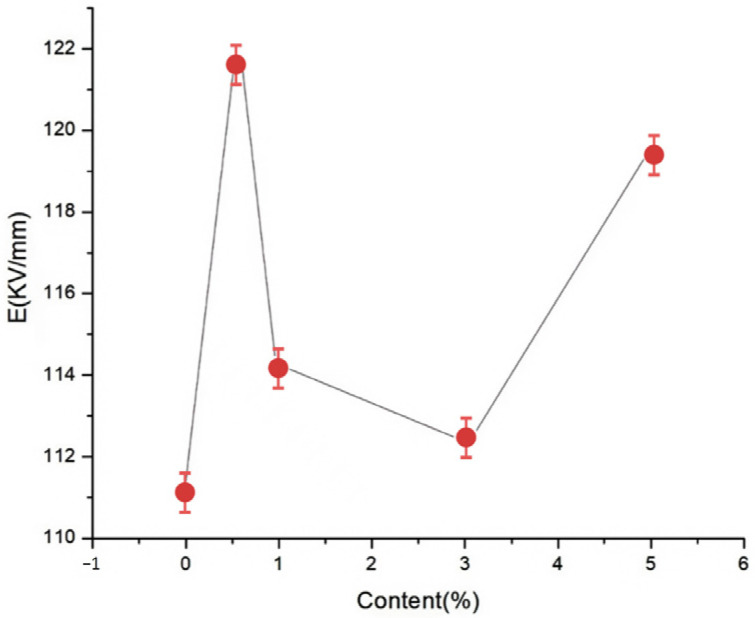
Weibull parameters for the different nano-MgO/LDPE composites during the power frequency breakdown tests.

To explore the effects of different nano-MgO particle contents on the composite breakdown field strength, nano-MgO/LDPE composites containing MgO loadings of 0.5, 1, 3, and 5% using the Weibull distribution were tested, and the results are presented in Figure 8 and Figure 9.

Figure 8 and Figure 9 show the Weibull distributions and Weibull parameters obtained for the nano-MgO/LDPE composites during the power frequency tests. It can be seen that upon increasing the MgO content, the breakdown field strength of the nano-MgO/LDPE initially decreased, then increased, and finally decreased again. The highest breakdown field strength was recorded for a MgO content of 0.5%, giving a value that was 1.8% higher than that of the pure LDPE. Under these conditions, the NPs were uniformly dispersed, resulting in the generation of a superior interfacial structure. In this case, the electronic transitions must overcome the higher carrier, which ultimately restrains directional electron migration. As a result, the number of free electrons in the interfacial region decreased. In addition, the electron scattering effect was strengthened, and the breakdown field strength clearly increased. Upon increasing the MgO content to 5%, the original network structure between the dopants was destroyed. Finally, the addition of a large number of NPs introduced additional impurities and polar bonds, thereby increasing the polar bond density of the composites.

### 2.6. PLM Test Results and Analysis

**Figure 10:**
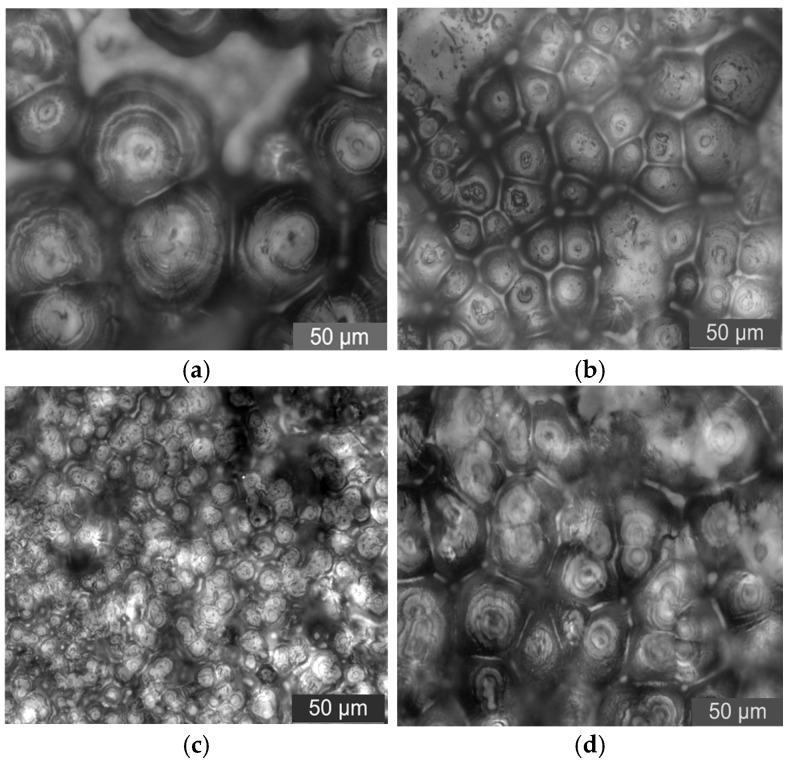
PLM patterns of nano-MMT/LDPE composites. (**a**) Pure LDPE. (**b**) PLM pattern of 1% MMT content sample. (**c**) PLM pattern of 3% MMT content sample. (**d**) PLM pattern of 5% MMT content sample.

**Figure 11:**
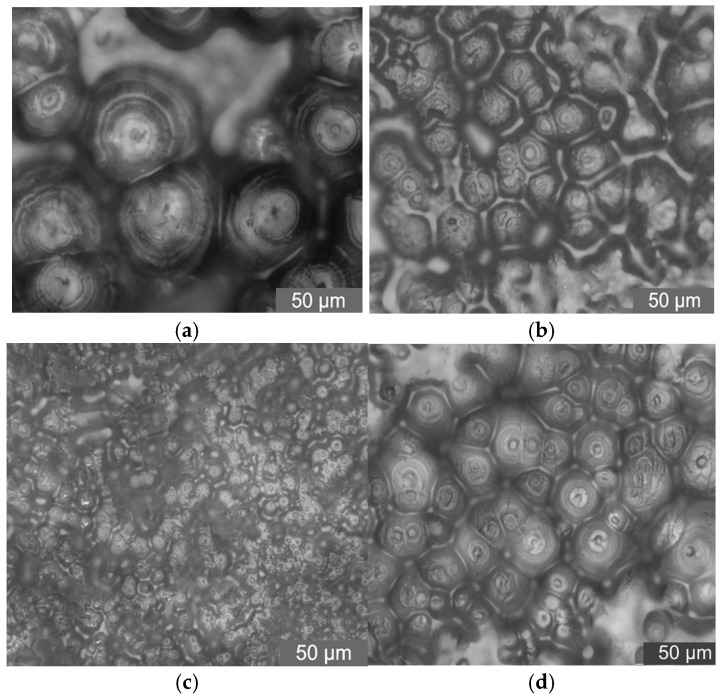
PLM patterns of nano-wollastonite/LDPE composites. (**a**) Pure LDPE. (**b**) PLM pattern of 0.5% wollastonite content sample. (**c**) PLM pattern of 1% wollastonite content sample. (**d**) PLM pattern of 5% wollastonite content sample.

**Figure 12:**
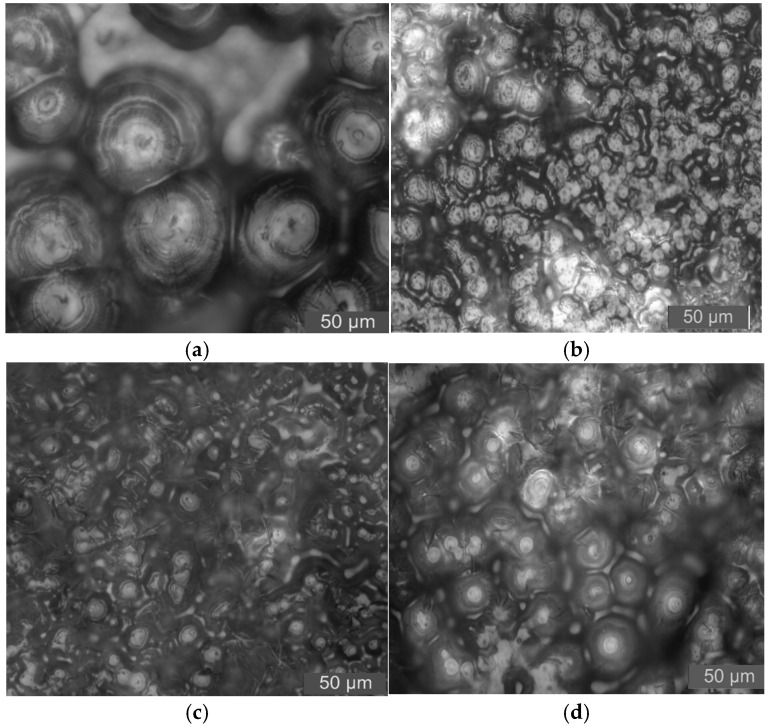
PLM patterns of nano-MgO/LDPE composites. (**a**) Pure LDPE. (**b**) PLM pattern of 0.5% MgO content sample. (**c**) PLM pattern of 1% MgO content sample. (**d**) PLM pattern of 3% MgO content sample.

In this test, PLM was used to observe the crystalline morphology and size of pure LDPE and different mass fraction nano-MMT/LDPE, nano-wollastonite/LDPE, and nano-MgO/LDPE. The effect of different mass fraction nanoparticles being added on LDPE crystallization is characterized. As such, the relationship between nanocomposites crystalline morphology with breakdown properties can be found. Among the materials, the 1%, 3%, and 5% MMT mass fractions of the nano-MMT/LDPE samples were selected, the 0.5%, 1%, and 5% wollastonite mass fractions of the nano-wollastonite/LDPE samples were selected, and the 0.5%, 1%, and 3% MgO mass fractions of the nano-MgO/LDPE samples were selected. The crystalline morphology observations are shown in Figure 10, Figure 11 and Figure 12.

From the test results shown in Figure 10, it can be seen that the addition of different mass fractions of nano-MMT particles produces different crystalline states of the composites. When the mass fraction is low, the sample’s internal crystal size is small. The structure is close. When the mass fraction of nano-MMT particles is 3%, the crystalline structure arranges closely. The crystalline size is uniform. With the increasing of the nano-MMT particles’ mass fraction, the sample’s crystalline structure increases gradually. The crystal nucleus arranges loosely. The heterogeneous nucleation effect of the nano-MMT particles weakens.

The PLM pattens of nano-wollastonite/LDPE samples are shown in Figure 11.

From the test results shown in Figure 11, it can be seen that the addition of nano-wollastonite particles decreases the polymer’s crystal size. Interfaces between spherulites are blurry. When the mass fraction of nano-ZnO particles is 1%, the sample’s crystal size decreases significantly. The crystal grains arrange closely. The areas of non-crystalline structure and interface structure increase. The internal crystalline morphology of the composite becomes complicated. With the increasing of nanoparticles mass fraction, the composite’s crystal size becomes larger. Combined with the breakdown test results above, it is verified that different crystalline morphologies and interface structures have different effects on composites’ breakdown properties [19,20].

The PLM pattens of nano-MgO/LDPE samples are shown in Figure 12.

From the results shown in Figure 12, it can be seen that the crystal size of the pure LDPE is large. There are larger non-crystalline regions between crystalline grains. Further, the crystalline grains arrange loosely. After nano-MgO particles are added, the crystalline grain sizes in the composites decrease in varying degrees. Among them, the crystal size of 3% nano-MgO/LDPE is the largest. The crystal size of 0.5% nano-MgO/LDPE is the least. Compared with pure LDPE, the crystalline grains’ arrangement in nano-MgO/LDPE composites is regular and tight. Thus, adding nano-MgO particles into a LDPE matrix plays the role of facilitating heterogeneous nucleation, which makes the LDPE crystalline morphology closer. The molecular segments contribute part of the physical crosslinking phenomenon [21,22].

### 2.7. Effects of NP Addition on the Composite Breakdown Properties

**Figure 13:**
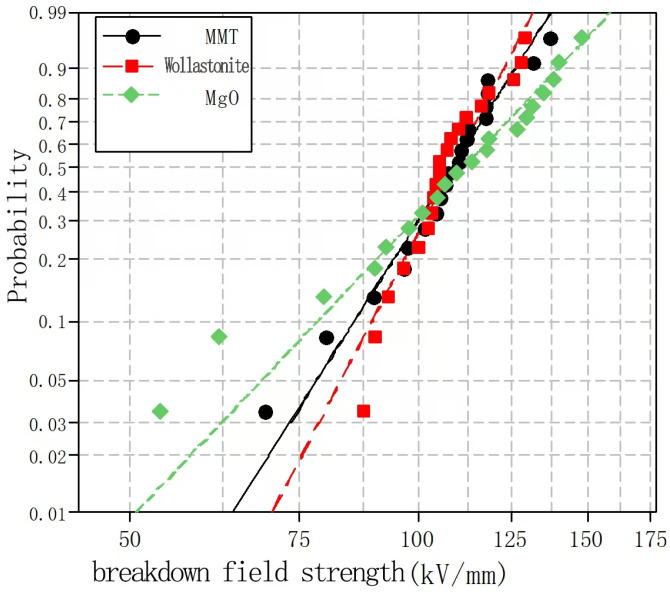
Weibull distributions of the power frequency breakdown characteristics of the different composites.

Samples 11, 6, and 1 were selected for a comparison of the breakdown properties of the three composites. The Weibull distributions of their corresponding power frequency breakdowns are shown in Figure 13.

As can be seen from Figure 13, the breakdown field strength of the nano-MgO/LDPE composite was higher than those of the nano-MMT/LDPE and nano-wollastonite/LDPE composites. This can be attributed to the fact that the dielectric strengths of most polymers are higher than those of the polymer matrix; this is related to the NP dispersity. Moreover, although the majority of NPs exhibit high dielectric constants and conductivities, they can cause local distortions in an electric field. In particular, in the case of spherical NPs, the degree of electric field distortion is more severe. Finally, the effect of the micromorphology on the breakdown strength was explored based on the observation of the electrical treeing paths. It was found that breakdown easily occurred at the spherulite interface but not in the crystals or amorphous areas. In this case, the amorphous structures included weakly and strongly amorphous regions, whereas the spherulite interface exhibited a weak amorphous structure. Owing to its low density, the amorphous area in the crystalline thin film possessed a high breakdown strength [23].

## 3. Materials and Methods

### 3.1. Composite Breakdown Tests

#### 3.1.1. Experimental Materials and Instruments

To prepare the composite materials, spherical nano-MgO NPs (Nanjing High Technology Nano Material Co., Ltd., Nanjing, China) with a particle size of 40 nm were employed, along with wollastonite (Nanjing High Technology Nanomaterial Co., Ltd., Nanjing, China), MMT (Lanzhou Petrochemical Company, Lanzhou, China), and LDPE (Borealis, Vienna, Austria). Antioxidant 1010 (Borealis, Vienna, Austria), anhydrous ethanol (Borealis, Vienna, Austria), and the PET films (Borealis, Vienna, Austria) were commercially available. The instruments employed during the composite preparation process are listed in Table 2.

#### 3.1.2. Composite Preparation

The composite samples were prepared and numbered as listed in Table 3. Prior to use, the LDPE, inorganic NPs, and antioxidant were dried in a vacuum oven at 80 °C for 12 h. Furthermore, before composite preparation, the drying oven was cleaned using pure LDPE to prevent residual substances in the mixing chamber from affecting the preparation quality. After heating the torque rheometer to 150 °C, the desired quantity of LDPE was added and the torque rate curve was observed. When the value reached a steady state, the inorganic nano-additive was added along with the antioxidant. Subsequently, the materials were blended at 150 °C using a screw speed of 40 rpm for 20 min. Notably, upon the addition of the inorganic powder to the polymer matrix, the mixture was stirred constantly to prevent nanoparticle agglomeration. The vulcanizing press was then preheated to 150 °C, and upon reaching this temperature, the desired composite sample was introduced and heated for 10 min. After this time, the pressure was increased every 5 min by 5 MPa increments until reaching 15 MPa. At this point, the sample was allowed to cool to room temperature in a cold press to prevent bubble generation. The sample thickness was ~100 µm.

## 4. Conclusions

In this study, nano-MgO, nano-wollastonite, and nano-montmorillonite (MMT) were employed as additives for doping into a pure low-density polyethylene (LDPE) matrix via melt blending to produce the corresponding nano-MgO/LDPE, nano-wollastonite/LDPE, and nano-MMT/LDPE composites. The breakdown field strengths of the different nanocomposites and the pure LDPE were evaluated and it was found that the inclusion of different nanoparticle (NP) types and contents affected the final composite breakdown field strength. More specifically, the highest breakdown field strengths of the composites were obtained when MgO, wollastonite, and MMT were added at doping levels of 0.5, 1, and 3%, respectively. Notably, the obtained values were 1.8, 7.5, and 6.7% higher than those of the pure LDPE, respectively. Furthermore, based on the Weibull distributions, it was deduced that the breakdown field strengths of the other composites did not increase significantly, likely due to NP agglomeration and the absence of uniform NP dispersions within the resin matrices. Notably, the nano-MgO/LDPE, nano-wollastonite/LDPE, and nano-MMT/LDPE composites were prepared simply and quickly in a sealed environment, thereby limiting the unintentional inclusion of impurities. Considering the different macroscopic- and micro-scale morphologies of MgO, wollastonite, and MMT, the thicknesses of the various layers constituting the multicore model were different, ultimately resulting in varying breakdown field strengths for the nanocomposites.

## Figures and Tables

**Table 1:** Theory of electric breakdown.

Theory	Explanation
Impact ionization theory	Under the effect of an electric field, the free electrons in the dielectric will be accelerated, gaining kinetic energy. Meanwhile, the collision lattice transfers the electric field energy to the lattice, which causes lattice vibration. The number of free electrons increases dramatically and the current also increases.
Avalanche breakdown theory	Due to the effect of impact ionization, an electron avalanche forms in the dielectric. For a constant pole distance, electron avalanche eventually leads to the destruction of the lattice structure and dielectric breakdown.
Charge trap theory	Electrons are injected into the polymer conduction band and are captured by traps within the composite. The electrons change from a high-energy state into a low-energy state and the redundant energy is transferred to another electron in the form of non-radiative energy to generate thermoelectrons. These thermoelectrons bombard the polymer molecule to generate free radicals and short segments, which increases the polymer free volume and promotes polymer breakdown.

**Table 2:** Instrumentation details.

Instrument	Model	Manufacturer
Vacuum oven	DZF-620	Shanghai Boxun Industrial Co., Ltd. (Shanghai, China)
Torque rheometer	RM-200A	Guangdong Airboo Electric Appliances Co., Ltd. (Shenzhen, China)
Vulcanizing press	XLB25-1	Huzhou Shuangli Automation Equipment Co., Ltd. (Huzhou, China)
Electronic balance	YP202N	Shanghai INESA Scientific Instrument Co., Ltd. (Shanghai, China)

**Table 3:** Raw materials.

Sample Number	Inorganic NP	Content (%)	Matrix Resin
0	None	0	LDPE
1	MgO	0.5	LDPE
2	MgO	1	LDPE
3	MgO	3	LDPE
4	MgO	5	LDPE
5	Wollastonite	0.5	LDPE
6	Wollastonite	1	LDPE
7	Wollastonite	3	LDPE
8	Wollastonite	5	LDPE
9	MMT	0.5	LDPE
10	MMT	1	LDPE
11	MMT	3	LDPE
12	MMT	5	LDPE

## Data Availability

Data are contained within the article.
